# Title X Policy Shifts and Michigan’s Reproductive Health Safety Net

**DOI:** 10.1001/jamanetworkopen.2025.22203

**Published:** 2025-07-21

**Authors:** Sarah D. Compton, Andrea Pangori, Audrey Widner, Kamilah Davis-Wilson, Sarah Wallett, Vanessa K. Dalton

**Affiliations:** 1 Department of Obstetrics and Gynecology, University of Michigan, Ann Arbor; 2 Planned Parenthood of Michigan, Ann Arbor

## Abstract

**Importance:**

The Title X Family Planning Program is a US federal grant program designed to offer comprehensive and confidential family planning and preventive health services to individuals in the US, regardless of their ability to pay. In 2019, a major change to the program—called the Final Rule—led to about 25% of participating clinics, including all Planned Parenthood affiliates, leaving the Title X program. Because Planned Parenthood of Michigan (PPMI) had served about 70% of Michigan’s Title X clients, its withdrawal from Michigan’s Title X program was anticipated to significantly alter the service coverage provided by the Title X program in the state.

**Objective:**

To examine the changes in geographic distribution, organizational profiles, and service delivery volumes of Title X health centers in Michigan—including those that left the program and then rejoined—before and after the implementation of the Final Rule.

**Design, Setting, and Participants:**

This cohort study included Title X–funded health centers in Michigan’s 83 counties from January 1, 2017, to December 31, 2023.

**Exposure:**

The enactment and reversal of the Title X Final Rule.

**Main Outcomes and Measures:**

Changes in service delivery volume, seen in (1) Title X penetration rate (number of unique females served at Title X–funded health centers), (2) PPMI penetration rate (number of unique females served by PPMI), and (3) reproductive health safety net penetration rate (sum of the females served by the Title X program and PPMI during the years it was excluded from Title X).

**Results:**

Title X service delivery counts decreased from 92 clinics before the implementation of the Final Rule in 2019 to 76 clinics during the Final Rule and increased to 92 after reversal of the Final Rule. Penetration rates were significantly higher before implementation of the Final Rule (incidence rate ratio [IRR], 3.98; 95% CI, 3.93-4.02) and after the Final Rule period (IRR, 2.96; 95% CI, 2.93-3.00). However, when PPMI service counts were included during the Final Rule period when PPMI was not formally part of Title X, there was considerably more stability in service delivery rates across the 3 periods (preperiod vs Final Rule period: IRR, 1.33; 95% CI 1.32-1.34; and postperiod vs Final Rule period: IRR, 0.99; 95% CI, 0.98-1.00). The mean PPMI penetration rate was only 1.41 times higher in the preperiod than in the Final Rule period (IRR, 1.41; 95% CI, 1.40-1.43).

**Conclusions and Relevance:**

This cohort study highlights PPMI’s role in the state’s Title X program and overall reproductive health safety net. During the relatively short time the Final Rule was in place, PPMI was able to maintain service delivery, protecting the public’s health against these regulations. It is not clear if PPMI, or similar organizations, could maintain service delivery over a longer period.

## Key Points

**Question:**

What were the changes to Michigan’s reproductive health safety net’s service delivery program associated with Planned Parenthood’s lack of participation in the Title X program during the Final Rule from 2019 to 2022?

**Findings:**

This cohort study found that there were fewer Title X clinics in Michigan during the Final Rule (92 before, 76 during, and 92 after) and significantly fewer women served by these clinics. However, because Planned Parenthood of Michigan continued to serve similar numbers of clients even while not participating in Title X, the reproductive health safety net was relatively maintained.

**Meaning:**

This study suggests that, because Planned Parenthood of Michigan was able to maintain service delivery levels over the short time they did not participate in the Title X program, the service delivery to the clients who rely on the reproductive health safety net in Michigan, as well as community health outcomes, were mitigated against the loss of federal funding.

## Teaser

This cohort study examines the changes in geographic distribution, organizational profiles, and service delivery volumes of Title X health centers in Michigan before and after the implementation of the Final Rule from 2019 to 2022.

## Introduction

Title X clinics are a critical source of affordable reproductive health care for many individuals in the US, including adolescents and those with low income. Historically serving over 4 million clients each year, the Title X Family Planning Program has been an important component of the health care safety net in the US,^1^ providing federally subsidized reproductive health services, including contraception and sexually transmitted infection screening. Title X clients are disproportionately members of racial and ethnic minority groups, those with low income, or uninsured individuals.^2^

Because the Title X program is administered by the US Department of Health and Human Services, the regulations and rules that govern the program often change with presidential administration transitions. For instance, various administrations have defined organizational funding eligibility, often specific to organizational activities related to abortion care. Although Title X funding cannot be used for abortion care, regulations have effectively been used to exclude organizations that provide abortion care—specifically, Planned Parenthood—outside the Title X program.^3,4^

Perhaps the most disruptive regulatory change occurred in 2019. Referred to as the Final Rule, this change went considerably further than prior regulations. The Final Rule made significant changes to the Title X program. The changes, often referred to as the *Title X gag rule*, included the following 3 key points. First, the new rule prohibited clinics that receive Title X funding from providing referrals for abortions. This prohibition marked a shift from previous policies, which required clinicians to offer neutral, factual, and nondirective information about abortion on request. Second, the rule required a more distinct separation, both physically and financially, between Title X–funded services and any abortion-related services. This requirement meant that facilities providing abortions would need to have separate locations and staff funded through non–Title X sources. Third, the changes prompted several health care organizations, including Planned Parenthood, to withdraw from the Title X program rather than comply with the new regulations. This withdrawal led to concerns about reduced access to reproductive health care, particularly for individuals with low income. These changes were part of the broader efforts by the presidential administration to restrict abortion access and reshape how family planning services are delivered in the US.

Immediately, about 25% of Title X grantees withdrew from the program nationally,^5^ including all Planned Parenthood affiliates. Michigan, like many other states, relied heavily on Planned Parenthood of Michigan (PPMI) to deliver Title X services. Prior to the Final Rule, 70% of Michigan’s program beneficiaries were served at PPMI.^6^ Consequently, the withdrawal of PPMI from Michigan’s Title X program was expected to markedly change the Title X service coverage in the state. The purpose of this study is to describe the changes to the geographic distribution, organizational profiles, and service delivery volumes of Title X heath centers, as well as the service delivery volumes of the general reproductive health safety net, in Michigan before, during, and after the Final Rule.

## Methods

We obtained data from the Michigan Department of Health and Human Services (MDHHS) Title X program, the US Census Bureau, and PPMI to describe the geographic location, organizational type of Title X sites, and service delivery counts in Michigan annually from January 1, 2017, through December 31, 2023. All study procedures were reviewed and deemed exempt from review by the University of Michigan institutional review board because it is a secondary analysis of anonymous data. This study followed the Strengthening the Reporting of Observational Studies in Epidemiology (STROBE) reporting guideline.

We used 3 time periods to frame our description of Michigan’s Title X program: (1) the preperiod (January 2017 to June 2019) comprises the years prior to the Final Rule enactment, during which PPMI was a Title X grantee; (2) the Final Rule period (July 2019 to December 2021), during which PPMI was not a Title X grantee, but continued to provide services in most locations; and (3) the postperiod (January 2022 to December 2023), during which PPMI had reentered the Title X program.

### Statistical Analysis

We used MDHHS data to identify the geographic location and organizational type of Title X health centers for our study years (2017-2023). Annual county characteristics (eg, population size, public health statistics, and social vulnerability index^7^—a measure that uses US Census data to determine the social vulnerability of every county and tract based on 15 social factors including poverty, lack of vehicle access, and crowded housing) were obtained from MDHHS and the US Census Bureau. These county characteristics were chosen based on the purpose of Title X: to increase access to reproductive health services—including sexually transmitted infection testing and treatment—for individuals with low income and those living in rural areas, regardless of their ability to pay. Changes in geographic location and demographic characteristics of counties with (and without) Title X health centers before and after the Final Rule was implemented were examined descriptively. We displayed demographic characteristics of Title X clinics for select years in each time period (2018, 2021, and 2023), as showing data from all years became overwhelming.

We used MDHHS and PPMI data to generate annual counts of the unique individuals serviced by the Title X program at each health center. To characterize changes in service delivery while accounting for population density, we adopted a previously developed measure^8^ referred to as a *penetration rate*. To quantify changes in the proportion of individuals with low income receiving reproductive health services, we calculated the penetration rate using the following: number of unique females served divided by number of females aged 15 to 44 years with low income in the service area, where low income was defined as 200% or less of the federal poverty level.

We calculated 3 penetration rates to describe service delivery volume changes: (1) Title X penetration rate, where the numerator is the number of unique females served at Title X–funded health centers; (2) PPMI penetration rate, where the numerator is the number of unique females served by PPMI; and (3) reproductive health safety net penetration rate, where the numerator is the sum of the females served by the Title X program and PPMI during the years it was excluded from Title X. The Title X and reproductive health safety net penetration rates were calculated at the MDHHS-provided Health Department Service Area level, except for the counties that were served by a single Title X site—either Planned Parenthood or a health department. The PPMI penetration rate was calculated at the county level. To visualize the patterns of penetration rate change over time, we created penetration rate biannual heatmaps from 2017 to 2023. To quantify the differences in the penetration rate between the 3 time periods while accounting for repeated measures across Health Department Service Areas and counties, we used mixed-effect logistic regression models. We ran separate models for each penetration rate (Title X, reproductive health safety net, and PPMI).

Data management and analyses were performed using R, version 4.3.2 (R Project for Statistical Computing), with extension packages tidyverse, version 1.3.1, tmap, version 3.3-4, tigris, version 2.1, ggh4x, version 0.2.8, and lme4, version 1.1.35.1. All *P* values were from 2-sided tests, and results were deemed statistically significant at *P* < .05.

## Results

Our analyses looked at Title X clinics within Michigan’s 83 counties, consisting of 14 MDHHS-provided Health Department Service Areas, 26 single county health departments, and 4 PPMI clinics. Three of the 14 MDHHS-provided Health Department Service Areas and 5 of the 26 single county health departments never had a Title X presence. Sixteen counties in Michigan had a PPMI clinic present during our study period (2017-2023). In addition, 2 of the MDHHS-provided Health Department Service Areas and 10 of the single county health departments had 1 or more PPMI clinics present. Title X service delivery sites decreased from 92 clinics before the implementation of the Final Rule in 2019 to 76 clinics during the Final Rule and increased to 92 after reversal of the Final Rule.

**Figure 1.**
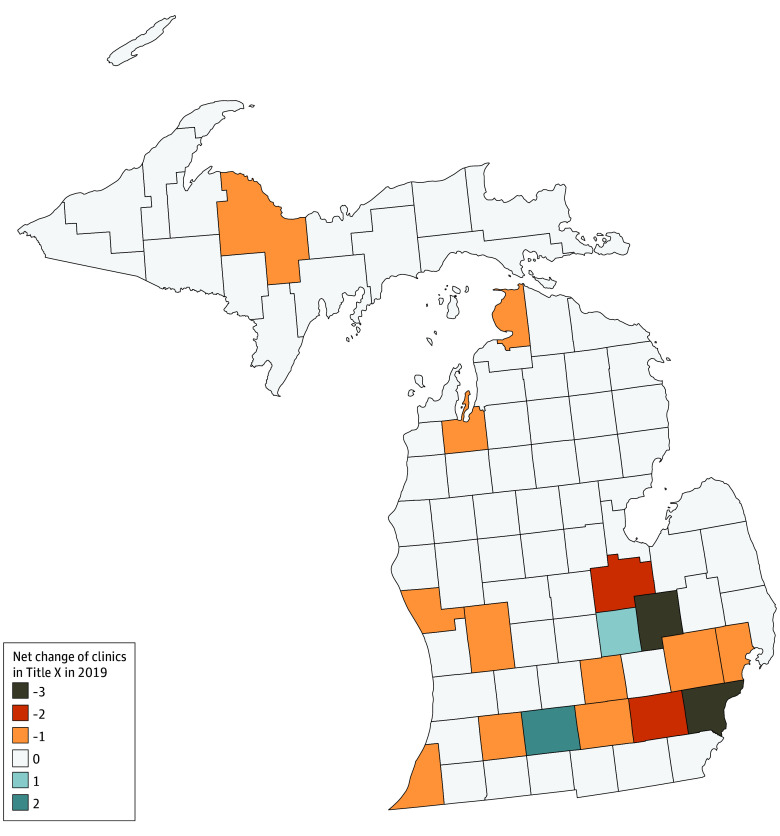
Net Change of Title X Clinics by County, 2019 This map captures the loss, absence of a loss or gain, or gain in the number of Title X clinics across all Michigan counties in 2019, when the Title X Final Rule was implemented.

Our analyses show that while there were major changes to Michigan’s Title X program, most PPMI clinics remained operational during the time they were not participating in the Title X program and service delivery counts decreased slightly from the years before the Final Rule was in effect. Nearly all the shifts in health center Title X funding occurred immediately after the enactment of the Final Rule; 23 of 25 clinics leaving the Title X program between 2017 and 2020 did so in 2019. Changes in the number and location of Title X–funded health centers in 2019 are shown in Figure 1.

The Table shows the percentage of counties with a Title X health center by select public health characteristics. For instance, during the preperiod, 38 of the 45 counties (84.4%) with a teen pregnancy rate above the national mean had a Title X–funded health center. This percentage decreased to 79.5% (31 of 39 counties) during the Final Rule period and remained close to that coverage level (29 of 36 counties [80.6%]) even after PPMI reentered the program. Similarly, prior to the Final Rule, 90.0% (8 961 339 of 9 957 488) of Michigan residents lived in a county with a Title X health center in 2018. While the Final Rule was in place, that proportion decreased to 78.5% (7 897 504 of 10 062 512), then increased to 89.0% (8 949 534 of 10 057 921) after the Final Rule was rescinded.

**Table.** Percentage of Counties With Title X Health Centers by Period and County Characteristics

County characteristic	2018^a^		2021^b^		2023^c^	
	Total counties, No.	Counties with a Title X clinic, No. (%)	Total counties, No.	Counties with a Title X clinic, No. (%)	Total counties, No.	Counties with a Title X clinic, No. (%)
In worst Social Vulnerability Index quintile^d^	17	14 (82.4)	17	15 (88.2)	17	15 (88.2)
Teen birth rate above the national mean	45	38 (84.4)	39	31 (79.5)	36	29 (80.6)
Rural location	62	49 (79.0)	62	50 (80.7)	62	51 (82.3)
Chlamydia rate above the national mean	10	10 (100)	10	6 (60.0)	9	8 (88.9)

^a^ Michigan residents living in a county with a Title X health center: 90.0%.

^b^ Michigan residents living in a county with a Title X health center: 78.2%.

^c^ Michigan residents living in a county with a Title X health center: 89.0%.

^d^ Social Vulnerability Index is a Centers for Disease Control and Prevention and Agency for Toxic Substances and Disease Registry place-based index to identify and quantify social vulnerability.

**Figure 2.**
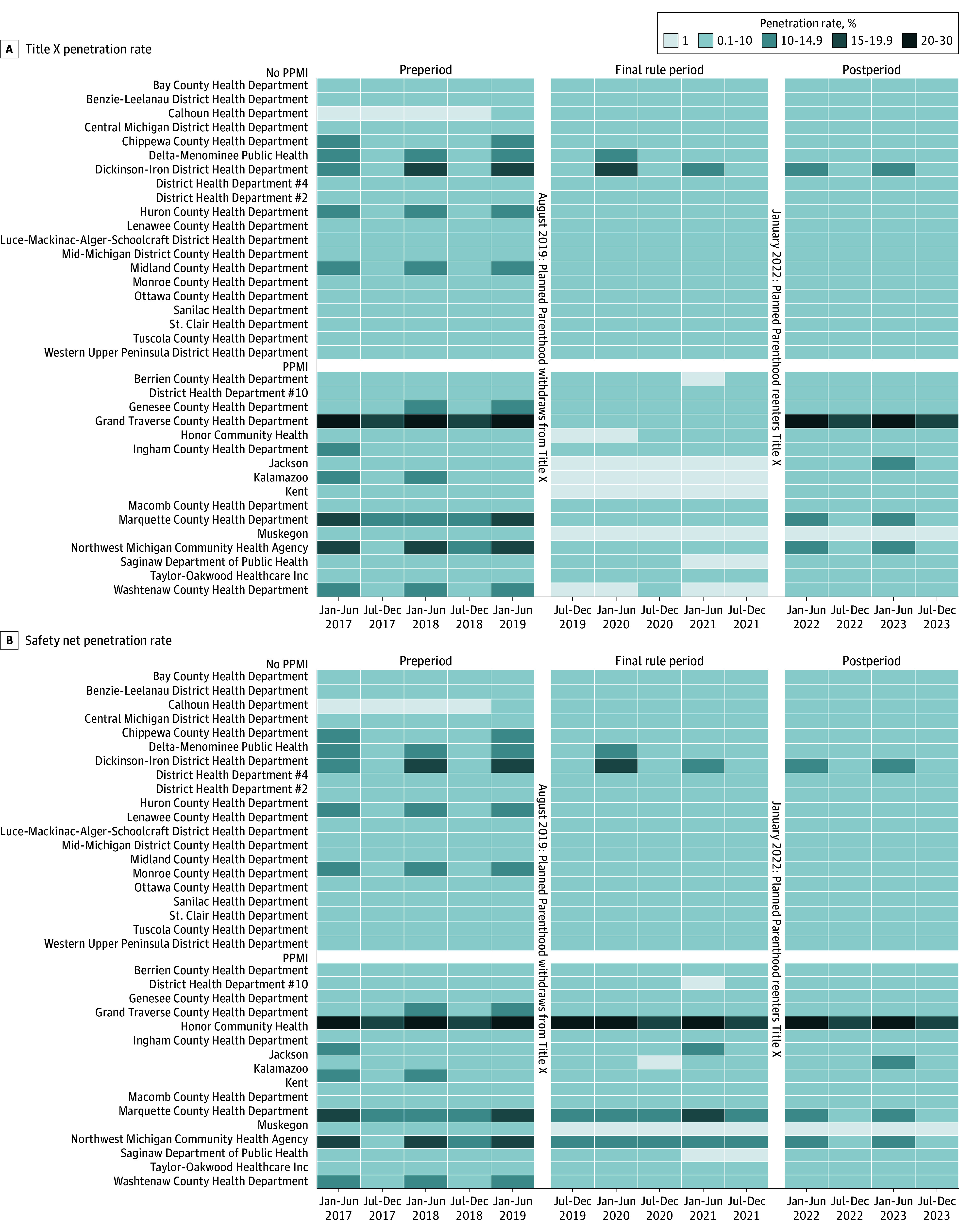
Title X and Reproductive Health Safety Net Penetration Rates With or Without Planned Parenthood of Michigan (PPMI) Service Delivery Activity, 2017-2023 Heatmaps illustrate biannual penetration rates from January 2017 to December 2023 for each Health Department Service Area (HDSA). Heatmaps are divided into preperiod (January 2017 to June 2019), Final Rule period (July 2019 to December 2021), and postperiod (January 2022 ti December 2023). A, Title X penetration rate. B, Reproductive health safety net penetration rate, which incorporates PPMI service delivery counts during the Final Rule period. HDSAs are categorized into 3 groups: no evidence of Title X clinic (not shown for visual display purposes), absence of a PPMI clinic, and presence of a PPMI clinic.

**Figure 3.**
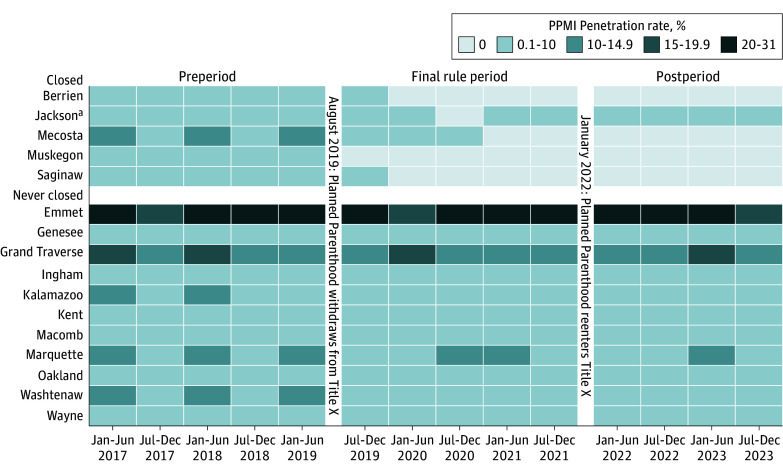
Planned Parenthood of Michigan (PPMI) Penetration Rates Over Time by County The heatmap represents biannual PPMI penetration rates from January 2017 to December 2023 for each county with a PPMI clinic and is separated into 3 time periods: preperiod (January 2017 to June 2019), Final Rule period (July 2019 to December 2021) when PPMI withdrew from Title X, and postperiod (January 2022 to December 2023) when PPMI reentered Title X. Counties are categorized into 2 groups: closed if the county’s PPMI clinics closed at any point during the study period and never closed if the county’s PPMI clinics remained open despite losing Title X funding. ^a^Jackson’s PPMI clinic closed and then reopened.

Figure 2 shows variation in penetration rate over time with and without adjusting for PPMI service delivery activity. When including only Title X–funded service counts, there was a marked reduction in the Title X penetration rate in Michigan between July 2019 and December 2021 (Final Rule period). We found that the mean penetration rate was 3.98 times higher in the preperiod than in the Final Rule period (incidence rate ratio [IRR], 3.98; 95% CI, 3.93-4.02) and 2.96 times higher in the postperiod than in the Final Rule period (IRR, 2.96; 95% CI, 2.93-3.00) (eTable in Supplement 1). However, when PPMI service counts were included (reproductive health safety net penetration rate), there was considerably more stability in service delivery rates across the 3 periods. We found that the mean penetration rate was only 1.33 times higher in the preperiod than in the Final Rule period (IRR, 1.33; 95% CI, 1.32-1.34) and that it was not significantly different in the postperiod than in the Final Rule period (IRR, 0.99; 95% CI, 0.98-1.00) (eTable in Supplement 1). Figure 3 shows the penetration rate over time for counties with at least 1 PPMI clinic, including clinics that closed during the study period. We found that the mean penetration rate was only 1.41 times higher in the preperiod than in the Final Rule period (IRR, 1.41; 95% CI, 1.40-1.43) and was 0.95 times lower in the postperiod than in the Final Rule period (IRR, 0.95; 95% CI, 0.94-0.96) (eTable in Supplement 1).

## Discussion

Our study describes changes to Michigan’s Title X program landscape and service delivery after the enforcement of the Final Rule, which led to the withdrawal of PPMI from the Title X program. The relatively small changes in reproductive health service delivery while the Final Rule was in place could be evidence of short-term resiliency of the reproductive health safety net in Michigan. Although these findings may appear reassuring, we also found evidence of an incomplete rebound after the Final Rule’s reversal. In other words, in some areas, the program may not be delivering services at the same level as in the pre–Final Rule period. It is not clear whether this decrease in service delivery rates is a result of declining demand or indicative of an increase in unmet need. In addition, work in other states has documented impacts to both patients and staff as a result of the Final Rule.^9^ To compensate for the loss of federal funding, some Planned Parenthood sites changed their fee schedules, passing additional costs onto clients, while staff were newly expected to carry out financial counselling with patients.

This study’s findings are important for at least 2 reasons. First, evaluations seeking to understand the public health effect of changes to the Title X program must account for short-term strategies deployed after funding losses that allowed organizations to continue service delivery. Second, we cannot assume that these strategies are sustainable beyond the short term. Failing to recognize that PPMI, and perhaps other organizations, managed to largely maintain service delivery levels in the short term could result in erroneously concluding that Title X family planning services are not meaningful for public health.

Although the reproductive health safety net in Michigan was largely maintained during the Final Rule period, not all communities fared the same. For instance, we found that even 2 years after PPMI reentered the program, nearly 10% of residents lived in counties that lost a Title X health center compared with 2018. Perhaps counter to expectations, access in rural areas was relatively preserved, with about 80% of rural counties continuing to have a Title X–funded clinic before, during, and after the Final Rule. This finding largely reflects the geographic location of Planned Parenthood health centers in Michigan, where health centers tended to be in more populated areas. Therefore, health departments and other clinics that remained in the Title X program during the time the Final Rule was in effect were more likely to be in rural areas. Furthermore, state decisions about where to locate health department Title X clinics may be influenced by service coverage by organizations such as Planned Parenthood.^10^ In complementary work, Smith and colleagues^11^ found that individuals belonging to racial and ethnic minority groups in Michigan and 9 other states were disproportionately represented in new contraceptive deserts after the Final Rule change.

### Limitations

This study has some limitations. First, we are not accounting for services delivered by other parts of the health care safety net such as community health centers, which do not receive Title X funding. In addition, the COVID-19 pandemic occurred at a similar time as our study period, and some of the changes in Title X service delivery could be due to pandemic-related closures and restrictions. Last, the Final Rule was implemented in August 2019, but because our data are biannual, the Final Rule period in our analyses begins in July 2019.

## Conclusions

In this cohort study of the reproductive health safety net, our study highlights the importance of PPMI in providing reproductive health services to individuals in Michigan who use and rely on Title X–funded clinics, even when PPMI was not part of the state’s Title X program. Although the Final Rule was the first time such a regulation was put into place, it likely will not be the last, and the fact that services have continued to lag behind the 2018 levels suggests it is easier to turn off services than to turn them back on. This finding could have major implications for public health.

## Notes

Supplement 1. **eTable.** Mixed Effect Logistic Regressions to Quantify the Difference in Penetration Rates Between the Different Time Periods for Each Subgroup (Title X, Reproductive Health Safety Net, and PPMI)

Supplement 2.
Data Sharing Statement
