# Supplementary material for: Title X Policy Shifts and Michigan’s Reproductive Health Safety Net
Source: JAMA Netw Open. 2025 Jul 21;8(7):e2522203. doi: 10.1001/jamanetworkopen.2025.22203 (PMC12281240; doi:10.1001/jamanetworkopen.2025.22203)
Supplement: Supplement 1. — eTable. Mixed Effect Logistic Regressions to Quantify the Difference in Penetration Rates Between the Different Time Periods for Each Subgroup (Title X, Reproductive Health Safety Net, and PPMI) [file jamanetwopen-e2522203-s001.pdf]

Supplementary Online Content

Compton SD, Pangori A, Widner A, Davis-Wilson, Wallett S, Dalton VK. Title X policy shifts and Michigan's reproductive health safety net. *JAMA Netw Open.* 2025;8(7):e2522203. doi:10.1001/jamanetworkopen.2025.22203

eTable. Mixed Effect Logistic Regressions to Quantify the Difference in Penetration Rates Between the Different Time Periods for Each Subgroup (Title X, Reproductive Health Safety Net, and PPMI)

This supplementary material has been provided by the authors to give readers additional information about their work.

© 2025 Compton SD et al. *JAMA Network Open.*

eTable. Mixed Effect Logistic Regressions to Quantify the Difference in Penetration Rates Between the Different Time Periods for Each Subgroup (Title X, Reproductive Health Safety Net, and PPMI)

Time Period	IRR	95% CI
Title X		
Pre-period vs Final Rule Period	**3.98**	**3.93-4.02**
Post-period vs Final Rule Period	**2.96**	**2.93-3.00**
Reproductive Health Safety Net		
Pre-period vs Final Rule Period	**1.33**	**1.32-1.34**
Post-period vs Final Rule Period	0.99	0.98-1.00
PPMI		
Pre-period vs Final Rule Period	**1.41**	**1.40-1.43**
Post-period vs Final Rule Period	**0.95**	**0.94-0.96**

Bold indicates statistical significance at the 0.05 alpha level
PPMI=Planned Parenthood of Michigan

© 2025 Compton SD et al. *JAMA Network Open.*
