# Supplementary material for: Title X Policy Shifts and Michigan’s Reproductive Health Safety Net
Source: JAMA Netw Open. 2025 Jul 21;8(7):e2522203. doi: 10.1001/jamanetworkopen.2025.22203 (PMC12281240; doi:10.1001/jamanetworkopen.2025.22203)
Supplement: Supplement 2. — Data Sharing Statement [file jamanetwopen-e2522203-s002.pdf]

Data Sharing Statement

Compton. Title X Policy Shifts and Michigan's Reproductive Health Safety Net. *JAMA Netw Open*. Published July 21, 2025. doi:10.1001/jamanetworkopen.2025.22203

Data
Data available: No
